# Retraction Note: Alleviation of nicotine-induced reproductive disorder, clastogenicity, and histopathological alterations by fenugreek saponin bulk and nanoparticles in male rats

**DOI:** 10.1007/s11356-026-37768-y

**Published:** 2026-04-21

**Authors:** Karima A. Hamed, Samia A. El-Fiky, Azza M. Gawish, Wagdy K. B. Khalil, Hanan R. H. Mohamed

**Affiliations:** 1 Department of Cell Biology, National Research Centre, 33 El-Bohous StDokki, P.O. 12622, Giza, 12622 Egypt; 2 Department of Zoology, Faculty of Science, Cairo University, Giza, 12613 Egypt

**Retraction Note: Environmental Science and Pollution Research (2022) 29:47488-47501**

10.1007/s11356-022-19123-z

The Editor-in-Chief and the Publisher have retracted this article. Following publication, concerns were raised about the data reported in Table 4, specifically regarding the mean and SEM values. The authors provided a response to the concerns raised and their raw data, however expert assessment concluded that this did not address the concerns raised satisfactorily and that the means and standard errors presented appear to be incorrect and that the statistical tests conducted are not appropriate. The Editor has therefore lost confidence in the results and conclusions of this article.

Authors Karima A. Hamed, Azza M. Gawish, Wagdy K. B. Khalil and Hanan R. H. Mohamed did not respond to correspondence from the Publisher about this retraction. The Publisher could not obtain current email address for author Samia A. El-Fiky.

